# Supplementary material for: Causal Relationship between Aging and Anorexia Nervosa: A White-Matter-Microstructure-Mediated Mendelian Randomization Analysis
Source: Biomedicines. 2024 Aug 16;12(8):1874. doi: 10.3390/biomedicines12081874 (PMC11351342; doi:10.3390/biomedicines12081874)
Supplement: Supplementary file 1 [file biomedicines-12-01874-s001.zip › Supplementary Figure/Figure S9/Leaveoneout plot of MR analysis for ubm-b-1846 to AN.pdf]

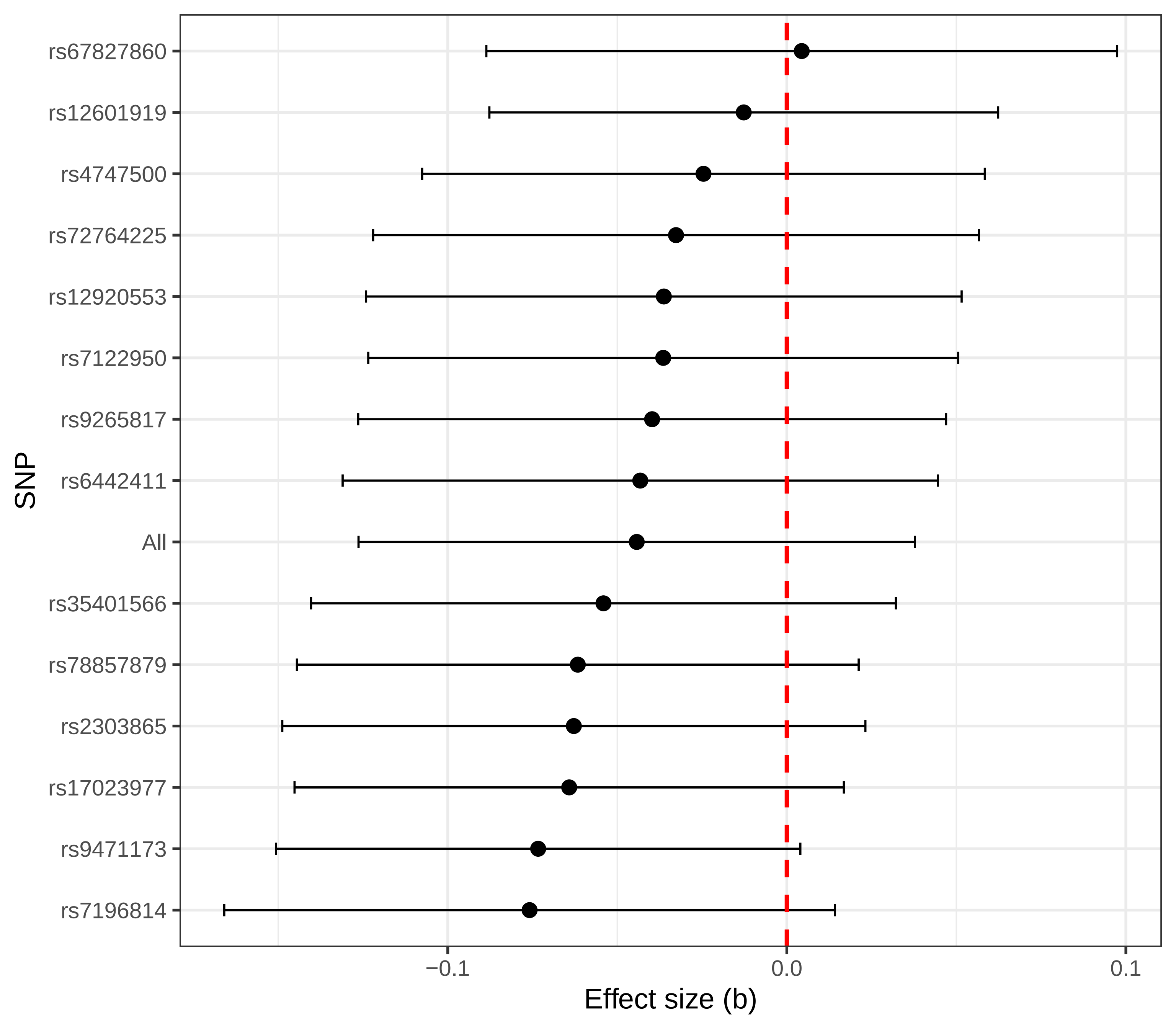
rs67827860
rs12601919
rs4747500
rs72764225
rs12920553
rs7122950
rs9265817
rs6442411
All
rs35401566
rs78857879
rs2303865
rs17023977
rs9471173
rs7196814
SNP
−0.1
0.0
0.1
Effect size (b)
